# Role of beta-adrenergic receptor subtypes in pig uterus contractility with inflammation

**DOI:** 10.1038/s41598-021-91184-5

**Published:** 2021-06-01

**Authors:** Barbara Jana, Jarosław Całka

**Affiliations:** 1 Division of Reproductive Biology, Institute of Animal Reproduction and Food Research, Polish Academy of Sciences, Tuwima 10 Str., 10-748 Olsztyn, Poland; 2 Department of Clinical Physiology, Faculty of Veterinary Medicine, University of Warmia and Mazury, Oczapowskiego Str. 13, 10-718 Olsztyn, Poland

**Keywords:** Immunology, Physiology, Diseases

## Abstract

Uterine inflammation is a very common and serious condition in domestic animals. To development and progression of this pathology often lead disturbances in myometrial contractility. Participation of β1-, β2- and β3-adrenergic receptors (ARs) in noradrenaline (NA)-influenced contractility of the pig inflamed uterus was studied. The gilts of SAL- and *E.coli*-treated groups were administered saline or *E.coli* suspension into the uterine horns, respectively. Laparotomy was only done in the CON group. Compared to the period before NA administration, this neurotransmitter reduced the tension, amplitude and frequency in uterine strips of the CON and SAL groups. In the *E.coli* group, NA decreased the amplitude and frequency, and these parameters were lower than in other groups. In the CON, SAL and *E.coli* groups, β1- and β3-ARs antagonists in more cases did not significantly change and partly eliminated NA inhibitory effect on amplitude and frequency, as compared to NA action alone. In turn, β2-ARs antagonist completely abolished NA relaxatory effect on these parameters in three groups. Summarizing, NA decreases the contractile amplitude and frequency of pig inflamed uterus via all β-ARs subtypes, however, β2-ARs have the greatest importance. Given this, pharmacological modulation of particular β-ARs subtypes can be used to increase inflamed uterus contractility.

## Introduction

The contractility of the myometrium (MYO) is very significant for reproductive processes such as the migration of embryos and its implantation in the uterus and parturition^1–3^. Disturbances in the physiological mechanism of MYO contractility lead to many disorders negatively affecting fertility. One pathology that develops as a result of impaired myometrial contractile activity (and/or immune processes) is uterine inflammation^4^. Endometritis and metritis are common disorders occurring in domestic animals caused mainly by bacteria. Uterine inflammation occurs especially after parturition, and difficult labor and fetal membrane retention predispose this pathology to expansion^5–7^. A mild course of inflammation evokes no serious disturbances in the course of estrous cycle. In more severe cases, however, occur the reproductive problems such as anestrus, repeat breeding, and not pregnant. Moreover, in the course of severe inflammation, there may be an increase in uterine contractility, which leads to vaginal discharge, or a reduction or loss of uterine contractility leading to the accumulation of inflammatory exudate in the uterine lumen^5,8–10^. The situations mentioned above are the most common reason for culling females in commercial herds worldwide, which reduces the profitability of production^5,7,8,13^.

Myometrial contractions and relaxations are regulated by the coordination and interaction of myogenic, hormonal and neurogenic stimuli^11–14^. The uterus in domestic animals is supplied by sympathetic, parasympathetic and sensory nerve fibers. However, in the uterus, sympathetic fibers are the most abundant and they exist in all layers of the uterine wall, and in the MYO they supply myocytes and blood vessels. The crucial neurotransmitter of this part of the nervous system is noradrenaline (NA)^15,16^ which (similarly as other catecholamines) exerts biological effects via adrenergic receptors (ARs). Taking into account both localization and functional activities, ARs are classified into α and β types. The α-ARs type includes subtypes: α1 and α2 (each with three isoforms). The β-ARs are represented by following subtypes β1, β2 and β3^17^.

With reference to the participation of NA in the contractility of healthy uteri, it is known that in various species this neurotransmitter stimulates (via α-ARs) and inhibit (via β-ARs) myometrial contractile activity^18–21^. Moreover, the role of β2- and β3-ARs in relaxation of human^22,23^, rat^24,25^ and pig^26,27^ MYO was indicated. In mice, the decrease in uterine contractility is mediated by β1- and β3-ARs^28,29^.

In the scope of sympathetic innervation of inflamed uterus, it is only known that *Escherichia coli (E. coli)*-induced uterine inflammation in the pig changes the populations of noradrenergic and non-noradrenergic caudal mesenteric ganglion neurons innervating the uterus^30^. Moreover, in the rats, general inflammation caused a reduction in the release of NA from myometrial nerve fibers and a drop in β-AR-mediated relaxation and an increase in α-AR-mediated contraction, and changes in ARs levels^31^. In the inflamed pig uterus, NA reduced the contractility^32^ and the expression of β (1, 2)- and α (1, 2)-ARs is altered^33^. It is also known that non-selective β-ARs blockers given preventively and therapeutically during the postpartum period in cows^34,35^ and sows^36–38^ increased uterine contractility, which favorably affected reproductive processes and production indexes. However, the meaning of particular subtypes of β-ARs in the contractile activity of inflamed uterus has not yet been described. An exact understanding of the receptor background of the NA influence can be important to the course and/or outcome of uterine inflammation. Therefore, a study of β1-, β2- and β3-ARs antagonists effects was carried out on NA-influenced contractile activity of the inflamed pig uterus.

## Results

### NA effect on the contractile activity

#### Myometrium

##### NA effect in comparison to the period before NA application

**Figure 1:**
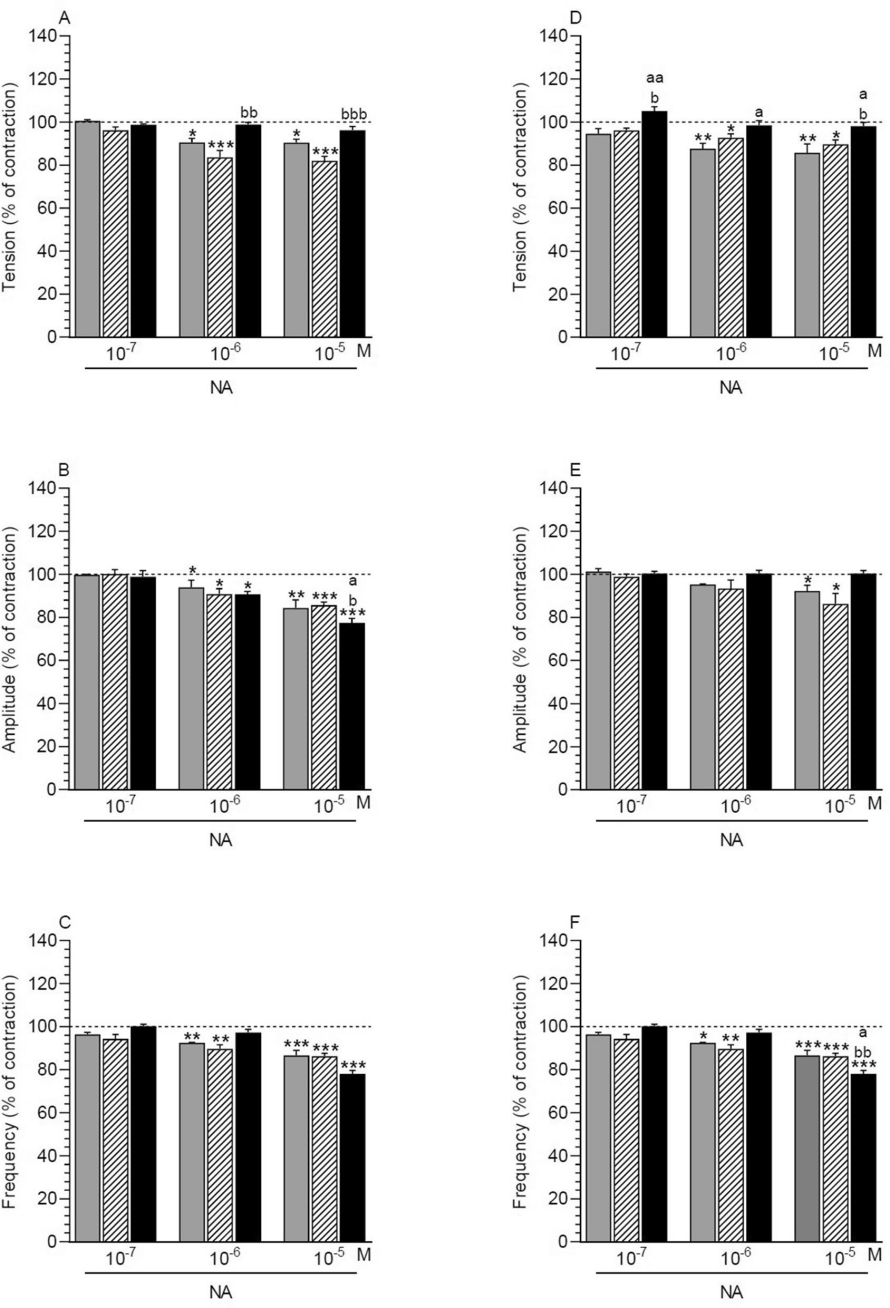
Effect of noradrenaline (NA) on the tension (**A**, **D**), amplitude (**B**, **E**) and frequency (**C**, **F**) of contraction in myometrium (**A**–**C**) and endometrium/myometrium (**D**–**F**) strips of gilts from the CON (grey bars), SAL (hatched bars) and *E. coli* (black bars) groups. Data obtained from five experiments (gilts, in each group). Effects of particular doses of NA are presented as a percentage (mean ± SEM) in relation to the basal (pre-treatment period) tension, amplitude and frequency, accepted as 100% (horizontal lines). *P < 0.05, **P < 0.01, ***P < 0.001—indicate differences in the comparison to the basal value in each group; ^a^P < 0.05—indicates a difference between the CON and *E. coli* groups for the same treatment; ^b^P < 0.05, ^bb^P < 0.01, ^bbb^P < 0.001—indicate differences between the SAL and *E. coli* groups for the same treatment.

NA (10^–6^, 10^–5^ M) significantly reduced the tension in MYO of the control and saline-injected uteri (Fig. 1A) and the amplitude in the three groups (Fig. 1B). A significant drop in the frequency in MYO of the control and saline-injected uteri was evoked by NA at a dose of 10^–6^ M and of the three groups by NA at a dose of 10^–5^ M (Fig. 1C).

##### Comparison of NA effect between groups

The tension in MYO of the inflamed uteri was significantly higher than in the saline-injected uteri after treatment with NA (10^–6^, 10^–5^ M) (Fig. 1A). The amplitude in MYO of the inflamed uteri was significantly lower in response to NA (10^–5^ M) vs. other groups (Fig. 1B). After adding NA, the myometrial frequency between the three groups did not differ significantly (Fig. 1C).

#### Endometrium/myometrium

##### NA effect in comparison to the period before NA application

In ENDO/MYO of the control and saline-injected uteri, NA led to a significant reduction in the tension (NA: 10^–6^, 10^–5^ M) (Fig. 1D) and amplitude (NA: 10^–5^ M) (Fig. 1E). Similarly, a significant drop in the frequency of ENDO/MYO was exerted by NA at a dose of 10^–6^ M in the control and saline-injected uteri and by NA at a dose of 10^–5^ M in the three groups (Fig. 1F).

##### Comparison of NA effect between groups

NA significantly increased the tension in ENDO/MYO of the *E. coli*-treated uteri vs. the control (NA: 10^–7^, 10^–6^, 10^–5^ M) and saline-injected (NA: 10^–7^, 10^–5^ M) uteri (Fig. 1D). After the use of NA (10^–5^ M), the frequency in ENDO/MYO of the inflamed uteri was significantly lower than in other groups (Fig. 1F). In the three groups, the amplitude was similar in response to NA (Fig. 1E).

### NA effect in the presence of β1-ARs antagonist

#### Myometrium

##### NA effect in the presence of antagonist in comparison to the period before these factors application

**Figure 2:**
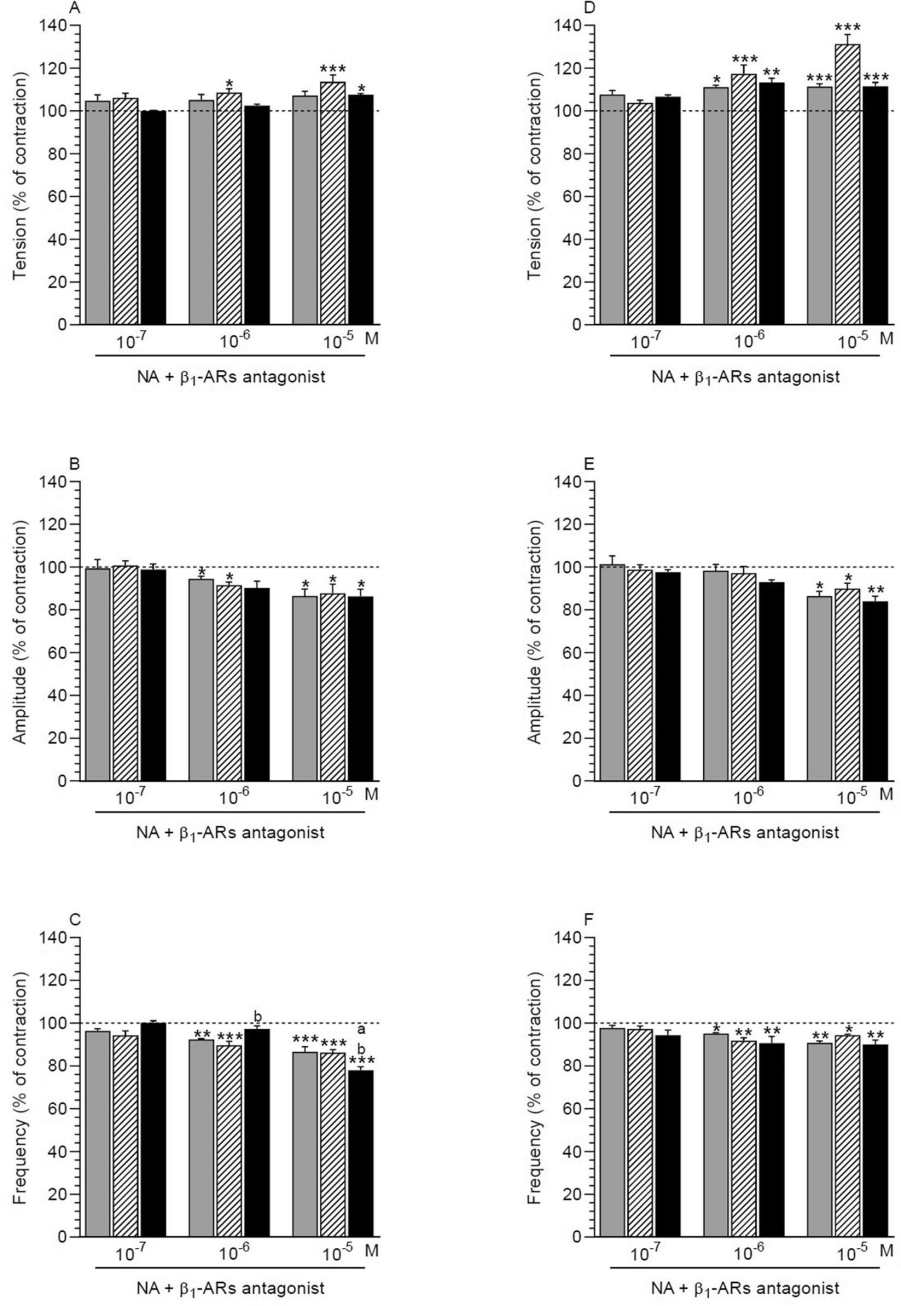
Effect of noradrenaline (NA) on the tension (**A**, **D**), amplitude (**B**, **E**) and frequency (**C**, **F**) of contraction in myometrium (**A**–**C**) and endometrium/myometrium (**D**–**F**) strips of gilts from the CON (grey bars), SAL (hatched bars) and *E. coli* (black bars) groups in the presence of β1-ARs antagonist. Data obtained from five experiments (gilts, in each group). Effects of β1-ARs antagonist and particular doses of NA are presented as a percentage (mean ± SEM) in relation to the basal (pre-treatment period) tension, amplitude and frequency, accepted as 100% (horizontal lines). *P < 0.05, **P < 0.01, ***P < 0.001—indicate differences in comparison to the basal value in each group; ^a^P < 0.05—indicates a difference between the CON and *E. coli* groups for the same treatment; ^b^P < 0.05—indicates a difference between the SAL and *E. coli* groups for the same treatment.

A significant rise in the tension in MYO was caused by β1-ARs antagonist and NA in the saline-injected (NA: 10^–6^, 10^–5^ M) and inflamed (NA: 10^–5^ M) uteri (Fig. 2A). This antagonist and NA significantly dropped the amplitude in MYO of the control and saline-injected (NA: 10^–6^, 10^–5^ M) and inflamed (NA: 10^–5^ M) uteri (Fig. 2B). A significant decreased in the frequency in MYO was evoked by antagonist with NA at a dose of 10^–6^ M in the control and saline-treated uteri and by antagonist and NA at a dose of 10^–5^ M in the three groups (Fig. 2C).

##### Comparison of antagonist and NA effect between groups

After treatment with β1-ARs antagonist and NA (10^–6^ M) the frequency in MYO of the inflamed uteri was significantly higher than in the saline-injected uteri (Fig. 2C). This parameter significantly lowered in the inflamed uteri vs. other froups after using antagonist and NA at a dose of 10^–5^ M. Antagonist and NA did not significantly alter the myometrial tension (Fig. 2A) and amplitude (Fig. 2B) in the three groups.

#### Endometrium/myometrium

##### NA effect in the presence of antagonist in comparison to the period before the application of these factors

In ENDO/MYO of the three groups, β1-ARs antagonist and NA significantly increased the tension (NA: 10^–6^_,_ 10^–5^ M) (Fig. 2D) as well as significantly reduced the amplitude (NA: 10^–5^ M) (Fig. 2E) and frequency (NA: 10^–6^, 10^–5^ M) (Fig. 4F).

##### Comparison of antagonist and NA effect between groups

After using β1-ARs antagonist and NA, all studied parameters in ENDO/MYO of the three groups did not differ significantly (Fig. 2D-F).

### NA effect in the presence of β2-ARs antagonist

#### Myometrium

##### NA effect in the presence of antagonist in comparison to the period before the application of these factors

**Figure 3:**
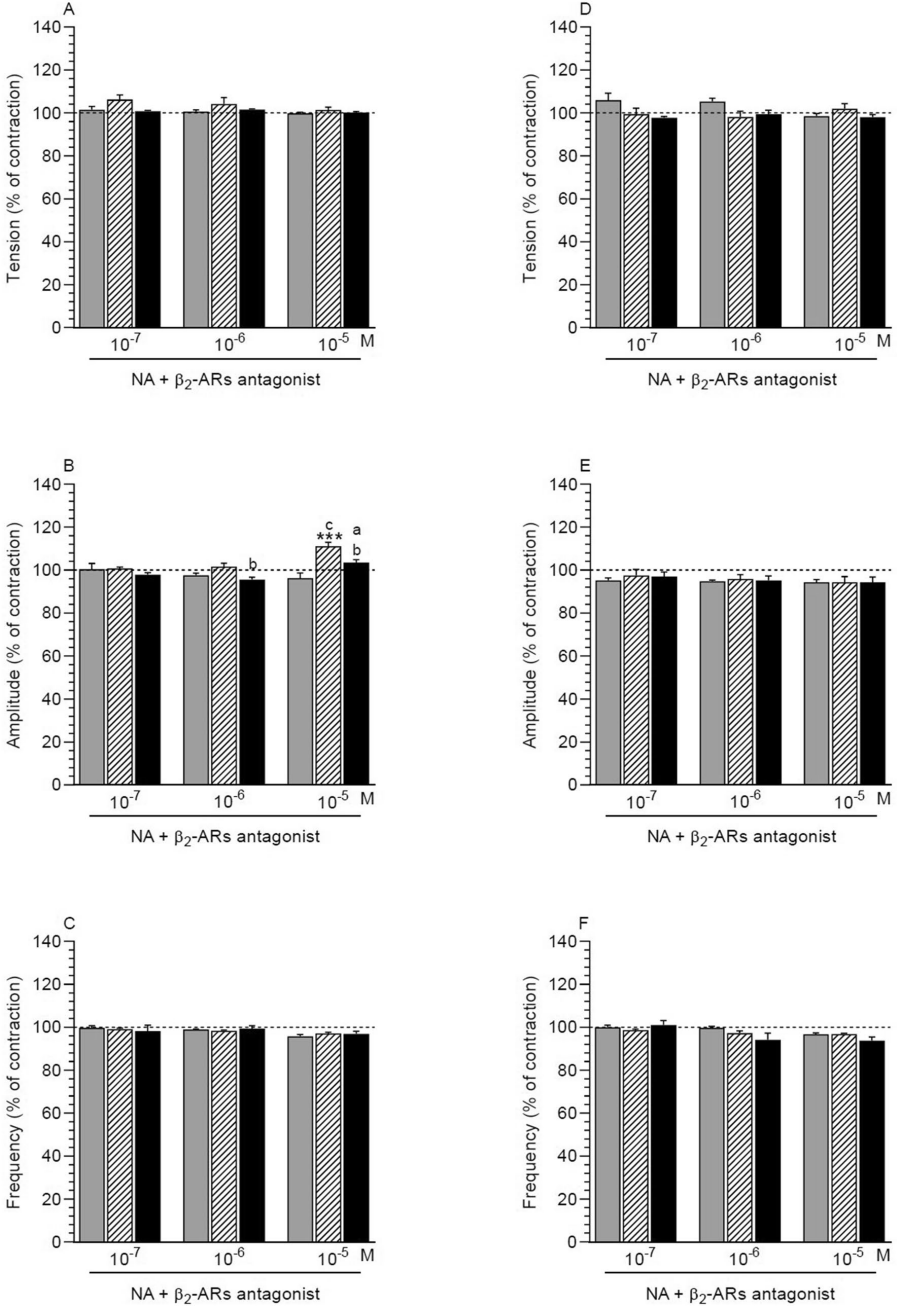
Effect of noradrenaline (NA) on the tension (**A**, **D**), amplitude (**B**, **E**) and frequency (**C**, **F**) of contraction in myometrium (**A**–**C**) and endometrium/myometrium (**D**–**F**) strips of gilts from the CON (grey bars), SAL (hatched bars) and *E. coli* (black bars) groups in the presence of β2-ARs antagonist. Data obtained from five experiments (gilts, in each group). Effects of β2-ARs antagonist and particular doses of NA are presented as a percentage (mean ± SEM) in relation to the basal (pre-treatment period) tension, amplitude and frequency, accepted as 100% (horizontal lines). ***P < 0.001—indicates a difference in the comparison to the basal value in each group; ^a^P < 0.05—indicates a difference between the CON and *E. coli* groups for the same treatment; ^b^P < 0.05—indicates a difference between the SAL and *E. coli* groups for the same treatment; ^c^P < 0.05—indicates a difference between the CON and SAL groups for the same treatment.

After addition of β2-ARs antagonist and NA (10^–5^ M), the amplitude in MYO of the saline-treated was significantly higher (Fig. 3B). These factors did not significantly change the tension (Fig. 3A) and frequency (Fig. 3C) in MYO of the three groups.

##### Comparison of antagonist and NA effect between groups

After using β2-ARs antagonist and NA (10^–6^ M), the amplitude in MYO of the inflamed uteri was significantly lower vs. the saline-treated uteri (Fig. 3B). The amplitude in MYO of inflamed uteri in response to the antagonist and NA (10^–5^ M) was significantly higher than in the control uteri and it was significantly lower vs. the saline-treated uteri. In the last uteri, this parameter was significantly increased compared to the control organs. The tension (Fig. 3A) and frequency (Fig. 3C) did not differ significantly between the three groups after using the antagonist and NA.

#### Endometrium/myometrium

##### NA effect in the presence of antagonist in comparison with the period before the application of these factors

Following administration β2-ARs antagonist and NA, all studied parameters in ENDO/MYO of the three groups were not significantly changed (Fig. 3D-F).

##### Comparison of antagonist and NA effect between groups

β2-ARs antagonist and NA did not significantly alter all studied parameters in ENDO/MYO of the three groups (Fig. 3D-F).

### NA effect in the presence of β3-ARs antagonist

#### Myometrium

##### NA effect in the presence of antagonist in comparison with the period before the application of these factors

**Figure 4:**
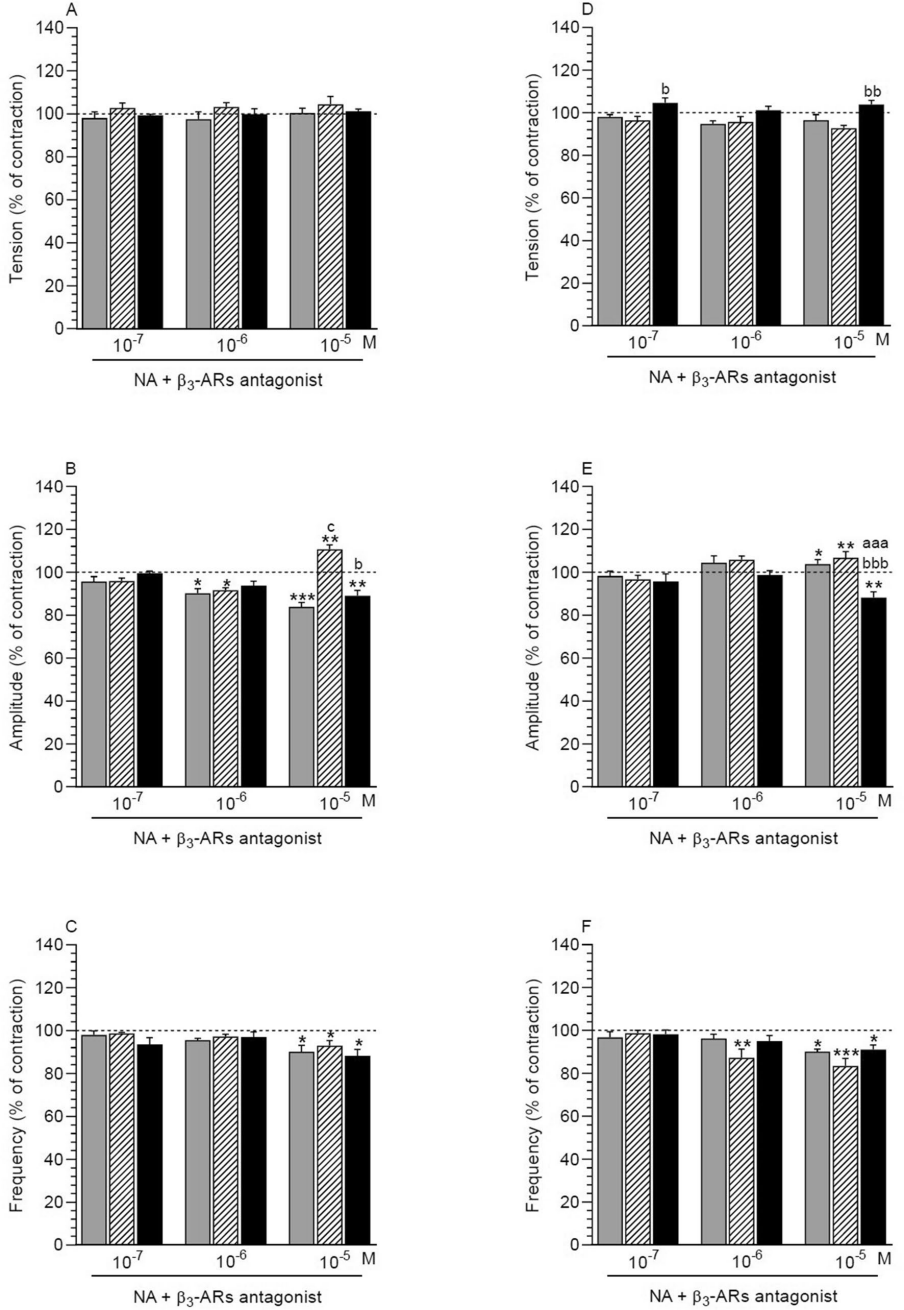
Effect of noradrenaline (NA) on the tension (**A**, **D**), amplitude (**B**, **E**) and frequency (**C**, **F**) of contraction in myometrium (A-C) and endometrium/myometrium (**D**–**F**) strips of gilts from the CON (grey bars), SAL (hatched bars) and *E. coli* (black bars) groups in the presence of β3-ARs antagonist. Data obtained from five experiments (gilts, in each group). Effects of β3-ARs antagonist and particular doses of NA are presented as a percentage (mean ± SEM) in relation to the basal (pre-treatment period) tension, amplitude and frequency, accepted as 100% (horizontal lines). *P < 0.05, **P < 0.01, ***P < 0.001—indicate differences in the comparison to the basal value in each group; ^aaa^P < 0.001—indicates a difference between the CON and *E. coli* groups for the same treatment; ^b^P < 0.05, ^bbb^P < 0.001—indicate differences between the SAL and *E. coli* groups for the same treatment; ^c^P < 0.05—indicates a difference between the CON and SAL groups for the same treatment.

In response to β3-ARs antagonist and NA a significant drop in the amplitude was noted in MYO of the control (NA: 10^–6^, 10^–5^ M) and inflamed (NA: 10^–5^ M) uteri (Fig. 4B). In MYO of the saline-injected uteri, β3-ARs antagonist and NA at a dose of 10^–6^ M significantly reduced the amplitude, while antagonist with NA at a dose of 10^–5^ M significantly increased this parameter. β3-ARs antagonist and NA (10^–5^ M) led to a significant drop in the frequency in MYO of the three groups (Fig. 4C). Antagonist with NA did not significantly alter the tension in MYO of the three groups (Fig. 4A).

##### Comparison of antagonist and NA effect between groups

After using β3-ARs antagonist and NA (10^–5^ M), the amplitude in MYO of the saline-injected uteri was significantly higher vs. other groups (Fig. 4B). The tension (Fig. 4A) and frequency (Fig. 4C) in MYO of the three groups were similar in response to β3-ARs antagonist and NA.

#### Endometrium/myometrium

##### NA effect in the presence of antagonist in comparison with the period before the application of these factors

β3-ARs antagonist and NA (10^–5^ M) significantly increased the amplitude in ENDO/MYO of the control and saline-injected uteri, while it significantly reduced in the inflamed uteri (Fig. 4E). In ENDO/MYO of the saline-injected uteri β3-ARs antagonist and NA (10^–6^ M) significantly reduced the frequency (Fig. 4F). A similar result was noted in the three groups after using antagonist and NA at a dose of 10^–5^ M. Antagonist and NA did not lead to significant changes in the tension in ENDO/MYO of the three groups (Fig. 4D).

##### Comparison of antagonist and NA effect between groups

The tension in ENDO/MYO of the *E. coli*-injected uteri was significantly higher after using β3-ARs antagonist and NA (10^–7^, 10^–5^ M) vs. the saline-injected uteri (Fig. 4D). Antagonist and NA (10^–5^ M) significantly lowered the amplitude in ENDO/MYO of the inflamed uteri compared to other groups (Fig. 4E). After application of these factors, the frequency in ENDO/MYO did not differ significantly between the three groups (Fig. 4F).

## Discussion

As mentioned earlier, the disturbances in the contractile activity of uterus may lead to origin, development and maintenance of inflammation (endometritis, metritis). In the *E. coli-*infected porcine uterus, the changes in prostaglandin (PG)F2α, PGE2, PGI2 and leukotriene (LT)C4 synthesis and release as well as the effect of PGI2, LTC4 and LTD4 on uterine contractility depend on the intensity and/or duration of the inflammatory process^32,39–41^. PGI2 and both LTs increased more the contractility of uterus with more intense acute inflammatory state and shorter duration^32,41^. This can result in the discharge of inflammatory exudate from the genital tract^5,39^. In turn, decreased myometrial contractility or its loss in the course of severe uterine inflammation are associated with the accumulation of mucopurulent exudate in the uterine lumen, what occurs mainly in cows^8–10^. In this regard, it is very important to know the mechanisms, including neurogenic, regulating contractility of the pathologically-changed uterus. Several studies have presented the role of (acetylcholine) ACh^42^, neuropeptide Y^43^, somatostatin^44^ and vasoactive intestinal peptide^45^ with the participation of their receptors in the neuronal regulation of inflamed uterus contractility. The effect of NA on the contractile activity of inflamed uterus^32,46^ as well as ARs expression in the pathologically-altered uterus^33^ have also been studied. In turn, the present study concerns the role of NA and particular subtypes of β-ARs in the contractility of the pig uterus with inflammation. The results of the macroscopic and histopathological estimation of the uteri utilized in this study have been previously reported and indicated the development of severe acute endometritis in organs treated with *E. coli*^33^. It should be added that the use of ACh to determine the contractile functionality of uterine strips to the present research confirms their viability and usefulness for further study. As compared to the period before ACh application, this neurotransmitter increased the amplitude and frequency in the CON and SAL and frequency in the *E. coli* group, while it reduced the amplitude in the latter group^42^. These observations are consistent with those presented previously in healthy and inflamed pig uteri^32,46^.

It is known that NA reduces the contractile activity of healthy pig uteri^3,19,20,26,27,32^ as well as other animal species^21,24,25,28,29^. The present study demonstrating the NA ability to decrease tension, amplitude and frequency in the control and saline-treated pig uteri compared to the period before this neurotransmitter addition, confirms the above findings. In analyzing changes in the uterine contractility under the influence of the same dose of NA, in some cases, different levels of reduction of the studied parameters between the CON and SAL groups were observed. This may be a derivative of changes in sensitivity and localization of β1-, β2- and β3-ARs after intrauterine saline administration. It was also reported that in the MYO of the CON and SAL groups used in the present study, the mRNA and/or protein expression of these receptors was not statistically different^33^. In the present experiment, the influence of an inflammatory state on NA-induced β1-, β2- and β3-ARs conducted uterine contractile activity was referred to both groups. However, significant differences in the values of the studied parameters were more frequently noted between the SAL and *E. coli* groups.

Similar to the CON and SAL groups, NA decreased the amplitude and frequency in an inflamed uterus but did not alter the tension, compared to the period before NA use. Moreover, in response to this neurotransmitter the amplitude and frequency in the inflamed uteri were reduced compared with healthy uteri. The authors’ previous studies showed that NA in the inflamed porcine uteri reduced the amplitude and frequency^32^ or increased the amplitude, but did not affect the frequency^46^. This situation may be due to changes in β- and/or α-ARs expression under the influence of an inflammatory process. In the present study we also revealed that the tension in the inflamed uteri was higher than in healthy uteri. It should be added that in the current study, for the first time, the NA action on the tension in a uterus with inflammation was examined.

Earlier, the role of β2- and β3-ARs in myometrial relaxation under physiological conditions in humans^22,23^, pigs^26,27^ and rats^24,25^ was reported. This effect in a mouse uterus is mediated by β1- and β3-ARs^28,29^. The use in the present work of the antagonists of particular β-AR subtypes revealed that NA reduces the uterine amplitude and frequency in the CON, SAL and *E. coli* groups and the tension in the CON and SAL groups through β1-, β2- and β3-ARs. Moreover, β1-ARs are involved in maintaining unchanged tension in the inflamed uteri in response to NA. At this point it is worth adding that the myometrial amplitude in the SAL group was higher vs. CON group after the use of the β2-ARs antagonist with NA as well as β3-ARs antagonist with NA. This situation may result from changes in sensitivity and localization of these receptors in response to intrauterine saline injections. Moreover, based on a greater elimination of the NA inhibitory effect on amplitude and frequency noted after blocking β2- than β1- and β3-ARs in the CON, SAL and *E. coli* groups, it was indicated that β2-ARs are crucial for NA action on these contractile parameters under physiological and inflammatory conditions. These findings concerning healthy uteri confirm earlier studies demonstrating that β2-ARs are a dominant subtype in the relaxing effect of NA on pig uterus during the estrous cycle and peri-implantation period^20,26^. In addition, the revealed role of β1-ARs in reducing the tension by NA in a healthy uterus (this study), supports the previous suggestion of the participation of this subtype in porcine uterine contractility^27^. It should be stressed that the present study, for the first time, demonstrated the participation of particular β-ARs subtypes in uterine contractility under inflammatory conditions.

As was mentioned above, the tension in an inflamed uterus in response to NA was increased, while the amplitude and frequency were reduced compared to healthy uteri. In MYO of the gilts from CON, SAL and *E. coli* groups used in the present study, the β1- and β3-ARs mRNA and/or protein expression were similar, but β2-ARs mRNA and protein expression were higher in the inflamed than in healthy uteri^33^. Thus, the drop in amplitude and frequency of the inflamed uterus in response to NA corresponded with the enhanced expression of β2-ARs. However, despite the higher content of these receptors in the MYO of a uterus with inflammation, the tension increased after NA treatment. As shows the present study, β1-ARs may be involved in the NA effect on this parameter. It is also possible that the NA influence on the contractile activity of inflamed uteri may also result from the different MYO localization of particular subtypes of β-ARs and their different ligand affinities. Moreover, NA may act indirectly on the contractile parameters of inflamed (also healthy) uteri by influencing the synthesis/release of substances affecting this function and NA influence can also be modulated. However, this supposition requires research. However, the existing data show the ability of NA to enhance (by β-ARs) the production of PGF_2a_ and PGI2 in the uterus^47,48^ and PGE2 in microglia^49^ as well as the cooperative interaction between NA and cysteinyl LTs to affect blood vessel contractility^50,51^. It should be added that these PGs and LTC4 and LTD4 are very essential for the contractile activity of inflamed pig uterus^32,41,46,52^.

It is known that catecholamines released under the effect of stressful factors have a negative influence on reproductive processes by the suppression of the muscular activity of reproductive organs. In cows, non-selective β-ARs blockers (propranolol—β1- and β2-ARs antagonist, carazolol—β1- and β2-ARs antagonist and β3-ARs agonist) improved uterine contractility parameters during prevention and therapy disorders of the post-partum period, including uterine inflammation^34,35^. The application of these blockers in sows shortens the duration of parturition and reduces the number of cases of mastitis-metritis-agalactia syndrome^36–38^. Considering the role of β1-, β2- and β3-ARs, including the dominance of β2-ARs in the NA effect on the particular contractile parameters of the porcine inflamed uterus found in the present study, this suggests the possibility of the preventive and therapeutic use of selective antagonists of particular ARs subtypes to more effectively block the uterine influence of catecholamines. This will allow more precise regulation of uterine contractile activity and, as a result, will contribute to improving the effectiveness of the prevention and treatment of post-partum diseases, such as placenta retention and uterine inflammation in domestic animals. It can contribute to the proper resumption of ovarian activity after parturition, and finally to improvement in fertility rates and a reduction of economic losses in farms.

## Conclusion

The data show that β1-, β2- and β3-ARs are involved in the decrease of the contractile amplitude and frequency of inflamed porcine uterus by NA. However, β2-ARs activation leads to relaxation of this uterus to a greater degree than activation of β1- and β3-ARs. Pharmacological modulation of particular β-ARs subtypes may be used to enhance myometrial systolic function during uterine inflammation.

## Materials and methods

### Animals

The all study procedures were approved by the Local Ethics Committee for Experiments on Animals (University of Warmia and Mazury in Olsztyn, Poland; Consent no. 65/2015). The guidelines in EU Directive 2010/63/EU for animals experiments were included. The experiment was conducted on fifteen gilts (Large White × Landrace, age 7–8 months, body weight 107.3 ± 1.8 kg /mean ± SEM/). A tester boar determined behavioral estrus. The gilts selected for the study did not show reproductive disorders: discharges from vagina were not visible and the second estrous cycle took place regularly. The animals were transported from a farm to the local animal house (University of Warmia and Mazury, Olsztyn, Poland) three days prior to testing. They stayed in individually pens (an area of about 5 m^2^ ) in conditions: temperature 18 ± 2 °C, natural daylight—14.5 ± 1.5 h, night—9.5 ± 1.5 h. The gilts received commercial diets and had access to water.

### Study design

After acclimatization, the animals were allocated randomly (on day 3 of the second estrous cycle—day 0 of the research), into following groups (five gilts in each group): *E. coli* group, with intrauterine administration of *E. coli* suspension*;* SAL group, with intrauterine administration of saline; CON group, only with a "sham" operation.

A detailed study procedures have as been reported previously^32^. Most importantly, following premedication (by the use of atropine, azaperone and ketamine hydrochloride) and general anesthesia (by the use of ketamine hydrochloride) median laparotomy was performed. Then, into each uterine horn in the *E. coli* group 50 ml of *E. coli* suspension (content: 10^9^ cfu per ml, bacterial strain O25:K23/a/:H1; Department of Microbiology, National Veterinary Research Institute, Puławy, Poland) were infused. In the SAL group, 50 ml of saline solution were infused. Bacterial suspension and saline solution were administered in 5 places (10 ml per each place) at a similar distance from each other. Horns were massaged to evenly distribute bacterial suspension and saline. In the animals of the CON group, only median laparotomy was done. All gilts tested were untreated in the period from surgery to euthanasia. On day 8 after surgery (the expected day 11 of the estrous cycle), animals were euthanized (an overdose of ketamine hydrochloride) and the uteri were collected. For examination of the contractile activity, fragments of the uterine horns collected from the middle part of the horns were placed on ice and transported to the laboratory within 5 min of collection.

#### Isolation of the uterine strips and recordings of isometric contractility

**Figure 5:**
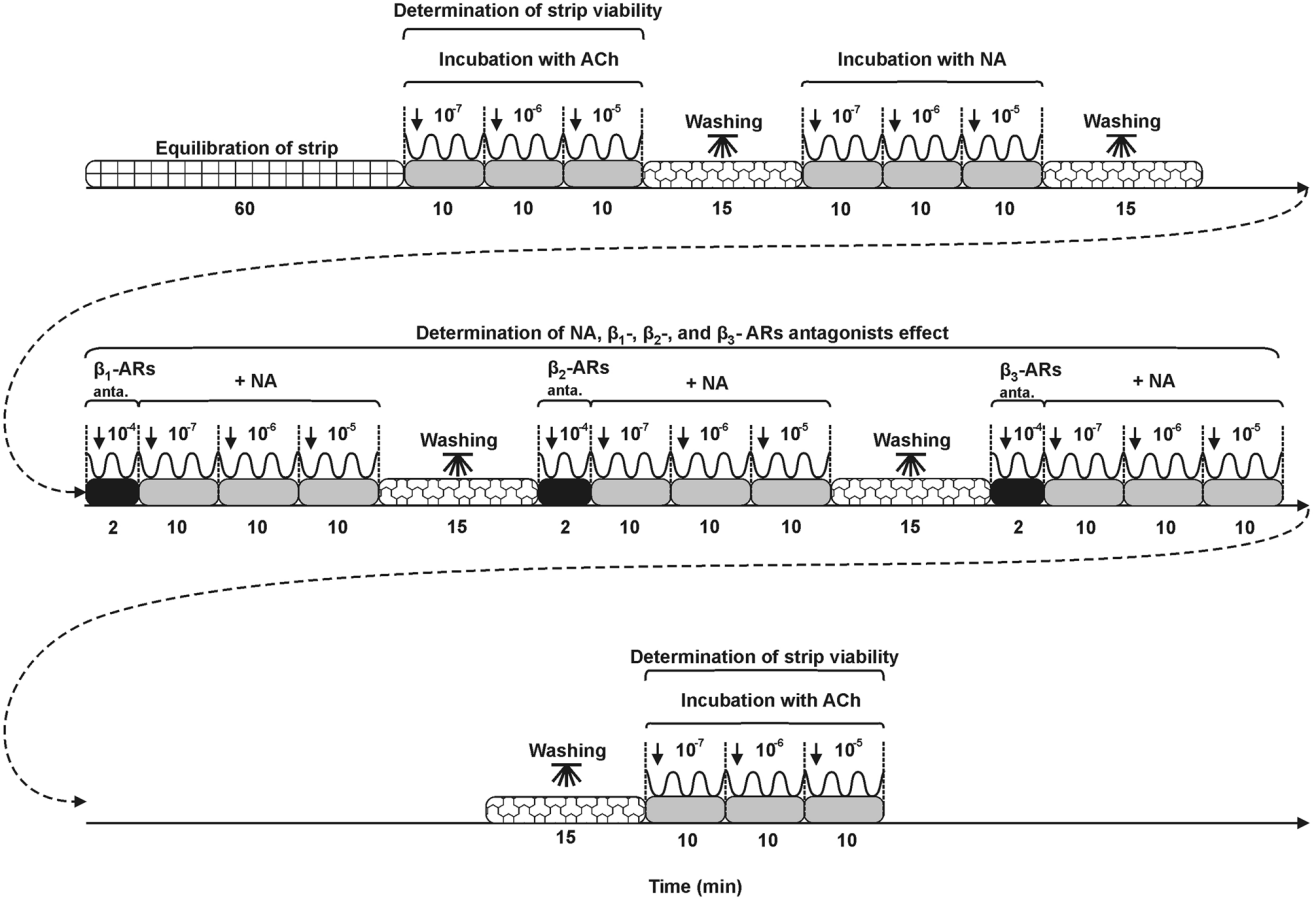
Diagram showing treatment of the uterine strips. Ach—acetylcholine; NA—noradrenaline; β1-ARs anta.—adrenergic β receptor subtype 1 antagonist; β2-ARs anta.—adrenergic β receptor subtype 2 antagonist; β3-ARs anta.—adrenergic β receptor subtype 3 antagonist. Concentrations of the used substances are given in moles.

To study the contractility, two kinds of strips (MYO and ENDO/MYO, size 5 × 3 mm) were excised from uterine horns^46^. The tissues were rinsed in saline and mounted between two stainless steel hooks in a 10-ml organ bath (Radnoti Unit Tissue Organ Bath System type 159920, Germany) at 5 mN resting tension. They were kept in Krebs–Ringer solution (mM/l: NaCl, 120.3; KCl, 5.9; CaCl_2_, 2.5; MCl_2_, 1.2; NaHCO_3_, 15.5; glucose, 11.5; pH 7.4) at 37 °C which was continuously oxygenated. The contractile tension, amplitude and frequency of tissues were measured with a force displacement transducer and recorded and analyzed in a computer using PowerChart software (Chart v5, scope v 5, AD Instruments). The scheme of uterine contractility measurement is presented in Fig. 5. After equilibration, the contractility of the strips was recorded for at least 60 min. Next, the contractile functionality of the strip throughout the study experiment was determined after ACh (Sigma, St. Louis, MO, USA) at doses of 10^–7^, 10^–6^ and 10^–5^ M. The ACh influence findings were given earlier^42^. After this, strips were treated with NA (Levonor, Warszawskie Zakłady Farmaceutyczne Polfa, Poland) at doses of 10^–7^, 10^–6^ and 10^–5^ M. The influence of each ACh and NA dose on the contractility was registered for 10 min. The part of results of NA action was reported earlier. This neurotransmitter was used to determine the contractile functionality of uteri^42^. The values of parameters in response to NA were also recorded in the presence of antagonists for β1- (RS-Atenolol, cat. no. 0387), β2- (ICI 118,551 hydrochloride, cat. no. 0821) and β3- (SR 59230A hydrochloride, cat. no. 1511) ARs, all from Tocris Bioscience. Regarding this, the strips were first under the influence of the β1-, β2- and β3-ARs antagonists at a dose of 10^–4^ M for 2 min., and then NA at doses of 10^–7^, 10^–6^ and 10^–5^ M was applied and the contractile activity was registered for 10 min. Between each study set, the tissues were washed three times in 15 ml of Krebs–Ringer solution. Finally, to re-evaluate the activity of the uterine strips, ACh was applied at doses as given as above. Any variability of less than 20% between measurements at the beginning and the end of the experiment was included in the statistical analysis. Doses of ACh and NA were reported in the literature^46,52^. Efficiency of β1-, β2- and β3-ARs subtypes antagonists doses were established in the initial research and confirmed by literature data^26,27^.

### Statistical analyses

The tension (resting/baseline tension expressed in mN), amplitude (the difference between the minimum and maximum value for a single contraction expressed in mN) and frequency (the number of observed peaks) of the uterine strips before the addition of the substances (pre-treatment period) and after their application (ACh at each dose 10^–7^, 10^–6^ and 10^–5^ M; NA at each dose 10^–7^, 10^–6^ and 10^–5^ M; NA at each dose 10^–7^, 10^–6^ and 10^–5^ M with β1-, β2- and β3-ARs antagonists at a dose of 10^–4^ M) were calculated for 10-min periods. Mean (± SEM) values of parameters calculated for particular groups before the addition of the substances were accepted as 100%. Effects of particular substances at each dose were regarded as a percentage (mean ± SEM) of the contraction tension, amplitude and frequency determined before their use. The Bonferroni test was applied to estimate the statistical significance between 1) mean values before (basal values) and after each treatment in each group, and 2) mean values between groups under the same treatment (ANOVA, InStat Graph Pad, San Diego, CA). Three thresholds for statistically significant differences were adopted: *P < 0.05, **P < 0.01, ***P < 0.001. No statistical power calculation was conducted prior to the study, and the determination of the size of animal experimental groups was based on data available in the previous studies, where the number of five animals in the case of pigs during uterine investigations are commonly accepted.

### Ethical approval

The studies presented in the manuscript were carried out in accordance with the ARRIVE guidelines. The all study procedures were approved by the Local Ethics Committee for Experiments on Animals (University of Warmia and Mazury in Olsztyn, Poland; Consent no. 65/2015). The guidelines in EU Directive 2010/63/EU for animals experiments were included. The datasets used and/or analyzed during the present experiment are available from the corresponding author upon reasonable request. Correspondence and requests for materials should be addressed to B.J.
